# Microbiome-based nutrition and the gut–muscle axis: emerging perspectives for athletic performance and recovery

**DOI:** 10.3389/fnut.2026.1927715

**Published:** 2026-09-07

**Authors:** Xinbao Zhang, Caijiao Sun, Baogen Yang

**Affiliations:** 1 Faculty of Physical Education, José Rizal University, Mandaluyong City, Metro Manila, Philippines; 2 Faculty of Arts, Krirk University, Bangkok, Thailand; 3 School of Physical Education, Qilu Normal University, Jinan, Shandong, China

**Keywords:** exercise adaptation, mitochondrial bioenergetics, polyphenols, post-biotics, prebiotics, probiotics, synbiotics

## Abstract

The gut microbiota has emerged as a significant regulator of exercise adaptation, recovery, and skeletal-muscle function, giving rise to the idea of the gut–muscle axis as a potential target in sports nutrition. This review summarizes current data on how microbiome-targeted nutritional approaches, such as probiotics, prebiotics, synbiotics, post-biotics, and polyphenol-rich dietary interventions, effect physiological processes related to athletic performance. Human research suggests that certain therapies may enhance endurance capacity, recuperation, gastrointestinal health, immunological function, and body composition, however, these advantages are typically strain-, formulation-, and population-specific. Further experimental evidence indicates that microbial metabolites, especially urolithins and short-chain fatty acids, may control anabolic signaling, inflammatory responses, mitochondrial function, and redox balance via pathways involving AMPK, PGC-1α, and associated molecular networks. However, there is still no direct validation of these pathways in human athletes, and the majority of mechanistic information comes from cellular and animal models. The translation of existing findings into evidence-based sports nutrition recommendations is further constrained by significant variation in study design, intervention characteristics, participant populations, and outcome measures. More robust longitudinal human investigations incorporating strain-resolved microbiome profiling, microbial metabolomics, and standardized physiological and performance outcomes are needed in order to identify effective intervention strategies and establish causal mechanisms.

## 1 Introduction

The dynamic interaction of training load, recuperation, diet, and biological resilience determines athletic efficiency. Although the majority of conventional sports science has focused on training scheduling and nutrition optimization, new research shows that the gut microbiota is an important but little-known element affecting performance results (1). The term “gut–muscle axis” describes the reciprocal relationship between the gut microbial ecosystem and skeletal muscle. Gut microbial communities may have an impact on immunological control, recuperation, and systemic energy balance while diet and exercise can alter the makeup and activity of microbes (2). Recent research suggests that athletes' gut microbiomes differ from those of sedentary people in that they are broader and more abundant in taxa linked to metabolic versatility and anti-inflammatory properties (3). These microbial compositions have been linked to faster recovery kinetics, decreased vulnerability to infections, and fewer gastrointestinal disruptions during training—all of which have a direct but substantial impact on performance. These results imply that the gut microbiota is not just a passive measure of health but is also linked to physiological changes seen in athletes. However, existing evidence does not prove that microbiome changes are a direct source of these adaptations as exercise, food, and other host factors may all contribute at the same time (4).

The gut-muscle link is supported by molecular plausibility. Short-chain fatty acids (SCFAs) and analogs of amino acids are examples of microbial metabolites that communicate with host signaling systems to affect inflammation tone, muscle protein turnover, and mitochondrial activity. Bioactive plant chemicals called polyphenols, which are found in large quantities in fruits, vegetables, and teas, additionally regulate this relationship by selectively enriching and functioning as prebiotic substrates (5). These connections establish a physiologically credible link among microbial diversity and quantifiable outcomes like strength, endurance ability and fatigue durability, supporting the use of microbiome-targeted methods in athletes (6). A growing number of studies are looking into the possibility of improving performance through nutritional modification of the gut microbiota. Probiotics, prebiotics, synbiotics, diets-high in polyphenols, and newly developed post-biotic formulations provide focused methods to enhance advantageous microbial taxa or functional products. Even though the current human studies indicate potential benefits, most report associations between microbiome-targeted interventions and selected physiological outcomes rather than showing that microbiome modulation directly drives gains in performance or recovery (7–9). A paradigm change in sports nutrition is evident in these results, which move away from broad dietary recommendations and toward targeted therapies that incorporate microbiota health as a key factor in training adaptability.

Notwithstanding its potential, a number of issues need to be resolved before microbiome research may be applied to sports nutrition. Small study populations, brief intervention periods, diverse strain selection, and inconsistent outcome evaluation limit the generalizability of current findings (10). Designing therapies that are universally successful is made more difficult by interindividual diversity in microbiome makeup, which is influenced by genetics, geography, training history, and typical food. The importance for thorough, long-term research that combines multi-omics assessments with standardized physiological and performance measurements is further highlighted by the lack of clear causal links between certain microbial alterations and performance results. By combining molecular understandings with translational implications, this review summarizes the state of the art on the gut-muscle axis in relation to sports performance. To give a thorough framework for nutritional approaches influenced by the microbiome, we will assess the current data, point out methodological flaws, and determine future research goals. These strategies have the ability to boost overall performance, maximize recuperation, and strengthen resilience, establishing the gut microbiota as a cutting-edge and useful aspect of modern sports research.

## 2 Conceptual framework of the gut–muscle axis

The gut-muscle axis is a complex two-way communication system in which skeletal muscle tissue and the intestinal microbiota interact metabolically in a dynamic interplay that largely affects metabolic balance, adaptations to training, and athletic output. The metabolic, endocrine, immunological, and neurological systems are all integrated into a single conceptual framework that describes how microbial diversity influences muscle functioning and how physical exercise influences the landscape of the gut microbiota (2, 4).

### 2.1 Bidirectional communication

In the suggested gut-to-muscle pathway, microbial metabolites may function as a bridge between intestinal microbial activity and skeletal muscle physiology. SCFAs, especially acetate, propionate, and butyrate, have been linked to the control of substrate metabolism, mitochondrial signaling, and host inflammation. SCFAs may affect AMPK, PGC-1α, and associated metabolic pathways, according to experimental studies. Nevertheless, there is still little proof that probiotics taken orally boost the creation of strain-specific SCFA, produce a particular systemic exposure, and then trigger these pathways in human skeletal muscle. Therefore, the SCFA–AMPK–PGC-1α sequence should not be considered a quantitatively established causal pathway in humans, but rather a plausible mechanistic model (6, 11).

**Figure 1:**
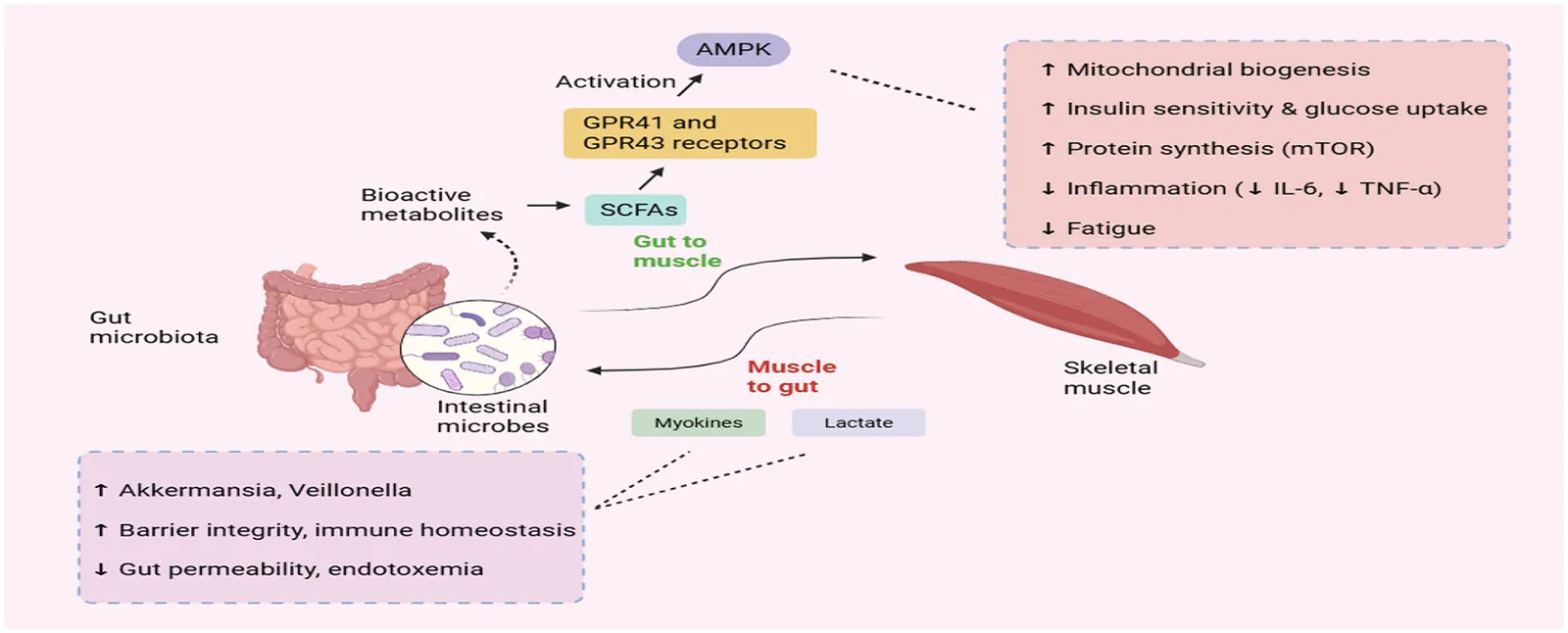
Bidirectional crosstalk between gut and muscle. Short-chain fatty acids derived from the gut activate GPR41/GPR43 and AMPK-related pathways, while lactate and myokines produced during exercise may affect the composition of gut microbes. SCFAs, short-chain fatty acids; GPR, G-protein-coupled receptor; AMPK, AMP-activated protein kinase; IL-6, interleukin-6; TNF-α, tumor necrosis factor-alpha.

Conversely, physiological alterations brought on by exercise significantly alter the gut microbiota. Exercise changes gut permeability, intestinal transit duration, mucosal blood flow, and the accessibility of bacterial fermentation substrates (12, 13). Myokines, such as apelin, irisin, interleukin-6 (IL-6), and interleukin-15 (IL-15), are released when skeletal muscle contracts. These myokines can alter immune system trafficking, gut barrier function, and enteroendocrine communication, which can favor particular microbial species (14, 15). Physical activity also changes the composition of gut microbes by increasing the generation of lactate, which provides a substrate for bacteria that use lactate, including Veillonella spp., and by modulating the metabolism of bile acids through improved enterohepatic circulation (3). This reciprocal link points to a biological feedback paradigm where the gut microbiota and regular exercise may have an impact on one another. However, the degree to which metabolites produced from the microbiome directly mediate human exercise adaptation is yet unknown (Figure 1).

A sophisticated, interconnected network of metabolic, hormonal, immunological, and neurological processes connects the gut to the muscles. Through metabolic processes, gut microorganisms create substances that the host can use as fuel and to control the way carbohydrates and fats are processed (2). From a hormonal perspective, these microbial byproducts can function similarly to hormones by stimulating receptors including FXR, PPAR, and TGR5 to affect metabolism and energy homeostasis in in general. Immunologically, the microbiota is constantly involved in immunological training and inflammatory regulation, which have a direct impact on muscle growth, repair, and recovery after workout (16). This communication also reaches the central nervous system via vagal signaling, microbial manipulation of neurotransmitter pathways, and interactions with neuronal circuits linked to motivation and weariness (4). These interconnected systems work collectively to generate a versatile, multimodal communication matrix that connects muscular function and gastrointestinal health.

## 3 Athlete microbiome: characteristics and exercise-driven adaptations

### 3.1 Distinct microbial signatures in athletes

A growing body of research suggests that the gut microbial communities of athletes are different from those of sedentary people, albeit these variations are impacted by training style, degree of competition, and dietary habits. Clarke et al. (17) carried out the first important study in this area on professional rugby players, who showed noticeably higher gut microbial diversity than non-athlete controls. A significantly wider taxonomic repertoire, including 22 phyla, 68 families, and 113 taxa, was found in athlete samples. Akkermansia, Ruminococcaceae, Succinivibrionaceae, Succinivibrio, Prevotellaceae, and Prevotella were more prevalent in athletes than in controls, but Bacteroides and Lactobacillus were less prevalent. Three different types of microbial communities were found in the gut microbiota of 33 competitive cyclists by Petersen et al. (18): a cluster that was dominated by Prevotella, a cluster that was dominated by Bacteroides, and a mixed cluster that included Bacteroides, Prevotella, Eubacterium, Ruminococcus, and Akkermansia. Interestingly, cyclists who trained for more than 11 h a week were far more likely to have higher Prevotella abundances (≥2.5%). In a similar vein, Bressa et al. (19) discovered that women who were physically active had greater levels of *Roseburia hominis, Akkermansia muciniphila*, and *Faecalibacterium prausnitzii* than women who were sedentary.

When higher-level and lower-level professional martial arts athletes were compared, Liang et al. (20) discovered that the former had considerably higher gut microbial richness diversity, as indicated by higher Shannon and Simpson diversity indices. In terms of taxonomy, athletes at higher levels had larger concentrations of Parabacteroides, Phascolarctobacterium, Bilophila, and Oscillibacter, whereas athletes at lower levels had higher concentrations of Megasphaera, Allisonella, and Citrobacter. In a multi-sport analysis of rowing (ROW), wrestling (WRE), and aerobics (AER), athletes had gut microbial profiles unique to their respective sports. Aerobics and rowing athletes differed from one another in 77% of genera, and aerobics and wrestlers differed from one another in 64%. Psychrobacter and Bacillus in male WRE and AER athletes, and Pseudomonas and *Eubacterium coprostanoligenes* group in female AER and ROW athletes, were important discriminant taxa (21). According to Kulecka et al. (22), there were differences in the gut microbial compositions of professional endurance athletes, esports gamers, and physical education students. At the species level, 45 bacteria distinguished athletes from esports competitors and 31 bacteria distinguished athletes from students. Particularly, Akkermansia species were more prevalent in athletes. According to the available data, athletes do not have a single universal microbiota. Instead, higher microbial diversity and repeated enrichment of taxa including Akkermansia, Prevotella, Veillonella, Faecalibacterium, Roseburia, Ruminococcus, and Eubacterium are frequently seen in athletic populations. However, significant inter-study variation suggests that microbial fingerprints are influenced by sport type, training intensity, competitive level, region, and dietary intake in addition to athletic status itself. Although higher microbial diversity in athletes has been repeatedly observed, the taxa found in different studies are not entirely consistent. This variation may be partially explained by variations in training volume, habitual diet, participant characteristics, geographic location, sport type, and microbiome assessment techniques. Therefore, the evidence does show a relationship between athletic status and microbiome makeup but there isn't a unique microbial profile that characterizes all athletes.

### 3.2 Effects of endurance and resistance training on gut microbial composition

Resistance and endurance training seem to affect the gut flora through distinct physiological demands. A study showed that quantifiable changes in the composition and function of gut microbes were brought about by 6 weeks of supervised aerobic exercise. A number of butyrate-producing taxa, such as Faecalibacterium, Roseburia, Lachnospira, unidentified Lachnospiraceae, and Clostridiales, were shown to be more abundant during exercise. Remarkably, after a 6-week sedentary washout period, many of these exercise-induced microbial alterations decreased (23). Munukka et al. (24) also discovered that in overweight women, 6 weeks of endurance exercise enhanced Akkermansia and decreased Proteobacteria. According to Morita et al. (25), in healthy older women, 12 weeks of aerobic exercise training focused on brisk walking increased the relative abundance of Bacteroides. The data for resistance training is more preliminary but still growing. According to a study, rats that received resistance training for 12 weeks had higher bacterial alpha richness and different gut microbial makeup. Coprococcus_1 was more prevalent during these changes, while Pseudomonas, Serratia, and Comamonas were less prevalent (26). According to Cullen et al. (27), *Roseburia faecis* and Roseburia genus abundance enhanced after 6 weeks of resistance training in young adults who were overweight or obese. *Faecalibacterium prausnitzii* also showed a considerable but non-significant increase. Additionally, Straub et al. (28) found that inactive adults' gut microbiomes changed in a time-dependent manner after 8 weeks of supervised resistance exercise. Faecalibacterium, *Roseburia hominis, Bacteroides massiliensis*, Lachnospiraceae, and Prevotellaceae-associated taxa were enriched after training, with *Roseburia hominis* exhibiting one of the most notable increases. Together, these studies show that gut microbial composition can be altered by both resistance and endurance training, while the precise taxa impacted depend on the research population, training duration, and exercise type.

### 3.3 Exercise intensity and microbiome remodeling

According to evidence from both acute exercise models and intensity-based training regimens exercise load can affect gut microbial composition, however, the degree and direction of change vary depending on host features, exercise intensity, and metabolic condition. A single session of moderate-intensity endurance exercise altered six gut bacterial species, according to Tabone et al. (29), although overall microbial diversity did not significantly change. Exercise reduced the abundance of Ruminiclostridium 9 and Clostridium phoceensis while increasing the abundance of Romboutsia, Blautia, Ruminococcaceae UCG-005, and *Escherichia coli* TOP498. According to Torquati et al. (30), low-active persons with type 2 diabetes experienced different gut microbiota responses depending on the intensity of their exercise. Higher concentrations of Bifidobacterium, *Akkermansia muciniphila*, Lachnospira eligens, Enterococcus species, and Clostridium Cluster IV taxa, such as Clostridium leptum and *Faecalibacterium prausnitzii*, were linked to moderate-intensity activity following training. On the other hand, *Methanobrevibacter smithii, Ruminococcus bromii*, and taxa belonging to the Erysipelotrichales and Oscillospirales orders were found in increased abundances during higher-intensity exercise. High-intensity interval training (HIIT) boosted gut microbial diversity and changed the distal gut microbial composition in obese mice, as shown by Denou et al. (31). An elevated Bacteroidetes/Firmicutes ratio and an enhanced Bacteroidales abundance were the hallmarks of these modifications. According to Yang et al. (32), mice's gut microbiota composition changed after moderate-intensity treadmill activity. When comparing the exercise group to sedentary controls, longitudinal study revealed significant increases in Bifidobacterium, Coprococcus, and an unidentified genus within the Clostridiales order. When considered collectively, the available data indicates that exercise intensity affects microbiome remodeling, with varying exercise loads favoring different bacterial taxa rather than resulting in uniform shifts in overall microbial diversity.

### 3.4 Gut barrier integrity during exercise

Numerous recent research have examined how biomarkers of gut barrier integrity are affected by acute strenuous exercise and exercise-related heat stress. Strenuous exercise raised I-FABP by about 400%−500%, according to an exploratory human investigation by Roca Rubio et al. (33). This increase was linked to systemic immunological and inflammatory alterations, suggesting that epithelium damage may be a factor in post-exercise inflammatory responses. Dziewiecka et al. (34) discovered that a 2,000-m ergometer test adversely altered gut integrity indicators in elite rowers, such as I-FABP, zonulin, LPS, and LBP, demonstrating that even brief, strong sport-specific exertion might momentarily impair intestinal barrier stability. Chantler et al. (35) showed that in healthy individuals, a single exercise session markedly enhanced intestinal permeability and markers of gut epithelial injury (I-FABP). Additionally, compared to exercise in thermoneutral circumstances, exercise in hot environments caused more gut damage reactions. According to Ogden et al. (36), exertional heat stress raised the levels of claudin-3 (CLDN-3) and intestinal fatty acid-binding protein (I-FABP) in the blood. Overall, these studies reported increases in biomarkers of intestinal epithelial damage and barrier integrity after acute strenuous exercise, although the precise indicators impacted varied among research populations and exercise regimens.

## 4 Biological pathways linking gut microbiota and skeletal muscle

This section's evidence comes from complimentary but different experimental environments. Human studies mainly assess physiological and performance outcomes while animal models and *in vitro* research offer molecular insights that cannot be directly applied to athletes. As a result, each category of evidence's results is interpreted differently in the ensuing subsections.

### 4.1 Microbial metabolites and energy metabolism

Microbial metabolites may affect skeletal-muscle bioenergetics by connecting dietary substrate availability, exercise-derived metabolites, and mitochondrial energy generation. After completing a marathon, Scheiman et al. (3) reported a boost in Veillonella abundance and discovered that genes related to lactate-to-propionate metabolism were enriched. Additionally, the researchers demonstrated that administering propionate reproduced the increased exercise phenotype. However, human athletes showed post-marathon enrichment of lactate-metabolism and Veillonella genes. On the other hand, intracolonic propionate injection and *Veillonella atypica* inoculation have been shown to improve performance in mice models. Therefore, even though these results indicate a biologically plausible gut-muscle mechanism but do not prove that the same causal pathway has been verified in humans. According to Lahiri et al. (37), skeletal muscle atrophy and decreased transcription of genes related to skeletal muscle growth and mitochondrial function were seen in germ-free mice. Short-chain fatty acid therapy partially corrected skeletal muscle deficits, while gut microbiota transplantation enhanced oxidative metabolic capacity and increased skeletal muscle mass. Acetate and succinate were found to be possible gut microbial metabolites by Ismaeel et al. (105), who also found that both metabolites enhanced skeletal muscle mitochondrial respiration during mechanical overload-induced hypertrophy. The researchers additionally stated that when given as post-biotics, acetate and succinate might enhance oxidative metabolism. All of these results point to a functional connection between skeletal-muscle metabolism and microbial metabolites in experimental models. They do not yet demonstrate that consuming probiotics causes a comparable metabolite exposure or mitochondrial reaction in human skeletal muscle. Furthermore, the concentrations attained by direct metabolite injection in experimental models could not accurately represent exposure in the skeletal muscle or circulation after food fermentation in humans.

### 4.2 Muscle protein turnover and anabolic signaling

Gut microbiota may support skeletal muscle preservation by controlling amino acid metabolism and anabolic signaling pathways involved in protein turnover. The majority of the evidence currently available for microbiota-regulated anabolic signaling comes from animal studies, with relatively little confirmation from human exercise research. Therefore, it is important to distinguish between mechanistic discoveries and effects that have been clinically shown in athletes. Germ-free mice showed skeletal muscle atrophy, reduced expression of insulin-like growth factor 1 (Igf1), and elevated expression of genes related to BCAA catabolism, according to Lahiri et al. (37). Gut microbiota transplantation enhanced skeletal muscle mass and decreased indicators of muscle atrophy. According to Valentino et al. (106), skeletal muscle adaptation to progressive weighted wheel running was hampered when antibiotics were used to alter the gut flora. Mice with microbiota disruption did not show the increases in satellite cell and myonuclear abundance seen in control animals, despite completing a similar training regimen. They also showed decreased hypertrophy of soleus and plantaris muscle fibers.

According to Aoi et al. (38), transplanting microbiota from exercise-trained mice enhanced AS160 and AMPKα phosphorylation in skeletal muscle, and recipient animals tended to have greater IGF-1 levels. These results imply that microbial communities that respond to exercise may have an impact on metabolic signaling pathways related to skeletal muscle adaptation. Liu et al. (39) showed that SCFA administration reduced the expression of atrophy-related genes, such as Atrogin-1 and MuRF1, while increasing AKT/mTOR phosphorylation and S6K1 expression. Sarcopenic mice showed increases in muscle mass, myofiber cross-sectional area, and muscle function in tandem with these molecular alterations. All of these animal investigations point to a potential connection between the gut microbiota and the control of anabolic signaling, muscle atrophy, and exercise adaption. Nevertheless, it has not been shown that microbiome-targeted supplementation in human athletes causes decreases in Atrogin-1 or MuRF1 and activation of AKT/mTOR signaling. Therefore, these results should be viewed as mechanistic data from experimental models rather than being proof of inhibited muscle breakdown in humans.

### 4.3 Redox homeostasis and oxidative stress control

Redox equilibrium plays a crucial role in both exercise adaptation and recuperation. Moderate production of reactive oxygen species (ROS) serves as a signaling mechanism that encourages training adaptations while excessive oxidative stress can lead to cellular damage, delayed recovery, and muscular exhaustion. By bolstering antioxidant defenses and shielding skeletal muscle from oxidative damage, metabolites produced from the gut microbiota may help maintain this equilibrium, according to new research. According to Otten et al. (40), in C2C12 myotubes, acetate, propionate, and butyrate raised intracellular glutathione (GSH) levels, suggesting improved cellular antioxidant capacity. Lu et al. (107) showed that sodium butyrate reduced heat stress-induced oxidative stress and preserved skeletal muscle homeostasis through autophagy-related pathways, indicating a more direct function for microbial metabolites in oxidative-stress control.

Sodium butyrate also reversed changes in Pax7, MyoD, and MyoG expression caused by oxidative stress in C2C12 cells, indicating the maintenance of myogenic regulation in the face of oxidative stress. These cellular results suggest that SCFAs may have an impact on antioxidant and myogenic signaling. Nevertheless, direct exposure of cultured muscle cells does not replicate the absorption, intestinal utilization, hepatic metabolism, and tissue distribution that occur after oral ingestion in humans. Therefore, it is not appropriate to interpret the efficacious doses employed in these tests as skeletal-muscle concentrations that can be attained with probiotic or dietary treatments. Human evidence is still scarce. Zhao et al. (41) found that supplementing with urolithin A (1 g/day) for 8 weeks reduced serum C-reactive protein (CRP) and superoxide dismutase (SOD) concentrations compared with placebo in a randomized, placebo-controlled trial involving male athletes who had received resistance training. However, there was no discernible difference in IL-6 between the groups. These results point to potential modulation of biomarkers related to oxidative stress and inflammation; however, since SOD by itself does not fully reflect redox homeostasis, they should be interpreted cautiously and require confirmation using more thorough evaluations of antioxidant status and oxidative stress. The majority of the mechanistic data has come from *in vitro* and animal studies, therefore human validation of microbiota-derived metabolites' potential role in redox control under experimental settings is still lacking.

### 4.4 Immune regulation and inflammatory responses

Skeletal muscle healing is significantly influenced by the immune response, which necessitates a carefully controlled balance between inflammatory activation and eventual resolution. The majority of the information that is now available about microbiota-mediated immune regulation during muscle healing comes from cellular research and animal models, and there are very few human studies that look at these particular processes during exercise. In a mouse model of cardiotoxin-induced acute skeletal-muscle injury, Hanna et al. (42) found that gut-derived, microbiota-dependent RORγ^+^ regulatory T cells aggregated in acutely wounded skeletal muscle, where they inhibited cells that produce IL-17A, encouraged the resolution of inflammation, and aided in tissue regeneration. In addition, Mann et al. (43) showed that IL-17A-producing Vγ6^+^ γδT cells quickly accumulated at the injury site, promoting early neutrophil recruitment, muscle stem/progenitor-cell proliferation, and subsequent muscle regeneration using a murine model of acute hindlimb skeletal muscle injury caused by cardiotoxin. Further studies comparing germ-free to specific pathogen-free mice and antibiotic-treated specific pathogen-free mice revealed decreased IL-17A production and γδT-cell accumulation, suggesting that this regenerative immune response was dependent on the microbiota. Martin et al. (44) investigated cultured rat L6 skeletal muscle myotubes at the cellular level. The cells were stimulated with lipopolysaccharide or palmitic acid after being exposed to acetate, propionate, or butyrate. The effects of palmitic acid stimulation differed depending on the concentration of SCFA: all three SCFAs decreased phospho-NF-κB p65, phospho-STAT3, MCP-1, IL-6, and RANTES at the higher concentration, while butyrate decreased numerous inflammatory responses at the lower concentration. These results show direct cellular effects in controlled experiments, but they do not prove that human skeletal muscle experiences similar SCFA concentrations or reactions to dietary or microbiome-targeted therapies. When combined, these cellular and animal studies offer mechanistic proof that microbiota-dependent immune cells and microbial metabolites can affect inflammatory processes related to skeletal muscle damage and regeneration. However, because these pathways have not been thoroughly proven in human athletes under exercise settings, their translational value is yet unknown.

### 4.5 Gut–brain–muscle communication

There is growing evidence that gut microbial signals may interact with brain circuits involved in physical activity and muscle function and influence exercise-related physiology. Dohnalová et al. (45) showed that microbiome-dependent fatty acid amides improved exercise performance in mice by stimulating CB1/TRPV1 sensory-neuron signaling, enhancing exercise-induced dopamine release in the ventral striatum, and boosting motivation for physical activity. According to Lahiri et al. (37), germ-free mice showed decreased expression of the neuromuscular-junction assembly genes Rapsyn and Lrp4, as well as decreased serum choline concentrations, along with decreased muscle strength and locomotor activity. Rapsyn and Lrp4 expression returned to normal when the gut microbiota was restored, indicating that microbial signals may support skeletal muscle neuronal control via neuromuscular communication pathways. The aforementioned mechanistic evidence is mostly derived from animal models used in experiments. Direct validation of these gut–brain–muscle connections in exercising humans is currently missing.

## 5 Microbiome-based nutritional modulation of the gut–muscle axis

Probiotics, prebiotics, synbiotics, post-biotics, and polyphenols are all discussed within the same broad framework since they may all affect the gut–muscle axis although they are not mechanistically or pharmacokinetically identical. While prebiotics supply the substrates that resident bacteria require, probiotics deliver live, strain-specific microorganisms, and synbiotics mix the two. Post-biotics include inanimate bacteria and/or their constituents while polyphenols go through human digestion, absorption, metabolism, and varied microbial alteration. As a result, results from one intervention category should not be immediately applied to another; instead, each should be assessed based on its unique composition, dose, metabolic fate, and athlete-relevant outcome.

### 5.1 Probiotics as ergogenic adjuncts

Probiotics, which are live microorganisms that provide health advantages when taken in sufficient quantities, have become an intriguing ergogenic supplement that may improve athletic ability via a variety of physiological routes. Enhancements in intestinal barrier strength, immune function regulation, improved recovery from exercise-related stress, and improved intake of nutrients and energy metabolism are all part of the mechanistic justification for a probiotic regimen in athletes (46). Probiotics have the ability to increase muscle mass through a number of interrelated processes. First, certain probiotic species improve intestinal barrier functioning by upregulating tight junction proteins, such as occludin, claudins, and zonula occludens-1 (ZO-1). This reduces intestinal permeability brought on by physical activity and the translocation of lipopolysaccharides (LPS), which causes systemic inflammation. This is especially important for endurance athletes, who often have temporary raises in intestinal permeability during extended exercise because of heat exertion and splanchnic hypoperfusion (46, 47). Second, probiotics collaborate with gut-associated lymphoid tissue (GALT), which makes up around 70% of the body's immune system cells, to modify immune activity. Certain strains, especially those of Lactobacillus and Bifidobacterium species, have been demonstrated to balance T-helper cell responses, boost secretory IgA production in the gut lumen, and boost the function of natural killer (NK) cells. These effects may lessen the incidence and intensity of upper respiratory tract infections (URTIs), which frequently plague athletes during times of intense exercise (48, 49). Third, positive exercise-related effects have been linked to specific probiotic strains. *Lactiplantibacillus plantarum* TWK10 enhanced endurance-related metrics and was associated with positive changes in body composition or muscle mass in both rodent and human trials (7, 50). The human studies, however, did not evaluate strain-specific metabolite synthesis, durable intestinal persistence, or if a unique gut-derived signaling route was responsible for the reported physiological alterations. Therefore, the clinical results support an ergogenic connection with TWK10 supplementation however the molecular mediators are yet unknown.

Direct as well as indirect ergogenic implications are supported by clinical data. In a double-blind RCT, healthy people who took *L. plantarum* TWK10 supplements for 6 weeks saw a substantial boost in muscle growth and endurance abilities (7). According to Jäger et al. (51), supplementing with B. breve BR03 and *Streptococcus thermophilus* FP4 decreased post-exercise inflammatory markers (lower IL-6) and preserved muscular durability and function after recuperation from muscle-damaging activity. Probiotic supplements may boost aerobic capacity, lower muscle-damage biomarkers like creatine kinase, and slightly improve physique composition in people that perform physical activity, according to a recent meta-analysis (52). According to additional information, probiotics significantly lessen the intensity of URTI symptoms overall and decrease the levels of TNF-α and IL-6 in professional athletes, which indirectly maintains performance through enhanced immunological resistance (53). Furthermore, compared to a placebo, using Lactobacillus plantarum TWK10 supplements for 6 weeks considerably enhanced endurance capabilities while preserving better blood glucose levels after intense exercise (54). In recreational endurance athletes, simultaneous multistrain pro/prebiotic treatment avoided exercise-associated rises in GI permeability and dramatically decreased circulating endotoxin units (55). According to Michalickova et al. (56), professional athletes who took Lactobacillus helveticus Lafti L10 supplements for 14 weeks saw a reduction in the number of symptoms and shorter course of URTIs, as well as an improvement in their self-rated vigor. When combined, these studies offer some evidence in favor of probiotic administration, especially for certain gastrointestinal, immunological, recovery, and endurance outcomes. The size of the benefits, however, varies depending on the strain, dose, length of the intervention, exercise model, and population studied, and they are not reliably replicated between research. Therefore, the results should be regarded as strain- and context-specific, rather than proving that probiotics have a generic ergogenic impact.

### 5.2 Prebiotics and synbiotics

Prebiotics are nutrients that have undergone selective fermentation, which alters the gut microbiota's composition and/or function and promotes the host's health. Prebiotics work as precursors that promote the development and metabolic function of helpful endogenous bacteria, especially those that produce SCFA, in contrast to probiotics, which introduce foreign microorganisms. Synbiotics are deliberate blends of prebiotics and probiotics intended to improve host biological outcomes and microbiome activity (57). SCFAs, mainly acetate, propionate, and butyrate, are produced during the breakdown of prebiotic fibers and have several uses related to sports performance. As the main source of energy for colonocytes, butyrate improves the strength of the gut barrier and lowers gastrointestinal permeability, which is important for endurance athletes who are suffering from gut stress brought on by physical activity (58). The liver is where propionate undergoes the majority of its metabolism. There, it aids in gluconeogenesis and may support blood glucose balance during extended workout. Acetate permeates the bloodstream and can be used as a means of energy by skeletal muscle and other peripheral tissues, especially when there is a shortage of carbohydrates (59).

**Figure 2:**
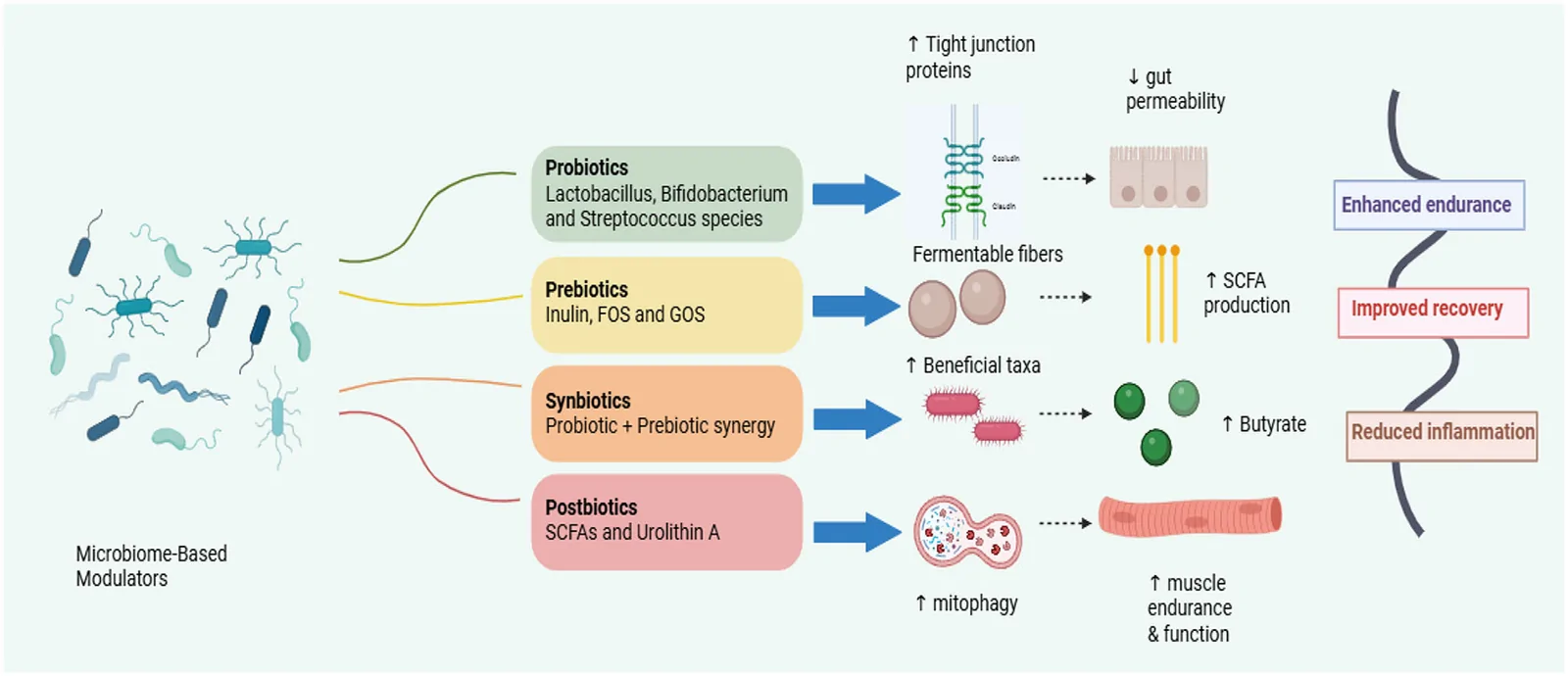
Microbiome-based nutritional approaches and their influence. The figure illustrates how gut microbiota and microbial metabolites are modulated by probiotics, prebiotics, synbiotics, and post-biotics, improving gut barrier integrity, decreasing gut permeability, boosting muscle performance, and improving exercise recovery and endurance. FOS, fructooligosaccharides; GOS, galactooligosaccharides.

Prebiotic supplementation affects host metabolism via a variety of mechanisms in addition to SCFA synthesis. Gut hormones that control hunger, glucose metabolism, and consumption of energy, such as glucagon-like peptide-1 (GLP-1) and peptide YY (PYY), are secreted in response to fermentable fibers (60). According to Stumpff and Manneck (8), prebiotic fermentation also lowers the colon's luminal pH, which can improve the uptake of minerals, especially calcium and magnesium, which are micronutrients essential for muscle contraction and recuperation. Although there isn't as much data to support prebiotic administration in athletes as there is for probiotics, new research points to possible advantages for recovery, metabolic health, and indirect performance improvement. In elite rugby players, using prebiotic supplements for 168 days decreased the frequency and intensity of digestive issues and decreased the duration of respiratory distress by 2 days. According to Parker et al. (61), it also enhanced salivary IgA production, indicating enhanced mucosal immune response. In endurance athletes, prebiotic treatment for 8 weeks decreased bacterial endotoxemia (sCD14) and exercise-heat-induced intestinal epithelial damage (I-FABP) (62). Additionally, prebiotic treatment (inulin + fructooligosaccharides) substantially boosted handgrip strength, a crucial measure of muscular function and frailty, in older persons in a randomized, double-blind clinical trial (108) (Figure 2).

Synbiotic compositions that combine particular probiotic strains with complementing prebiotic substrates are an appropriate approach to optimize ergogenic potential and microbiome modulation. By supplying the advantageous bacteria and the materials required for their longevity, growth, and metabolic processes in the competitive gut ecosystem, the synbiotic strategy theoretically offers benefits above each element alone (63). Mechanistically, synbiotics reduce endotoxemia and enhance nutrient signals by increasing the number of butyrate-producing taxa (Faecalibacterium, Roseburia) and inhibiting pro-inflammatory bacteria. By increasing mitochondrial function and decreasing catabolic cytokine communication, these processes result in better muscular function and recuperation capacity (64). In comparison to probiotic, omega-3, or placebo alone, synbiotic treatment in conjunction with USRPT enhanced elite sprint swimmers' explosive, high-velocity upper-body endurance (65). In male football players, 6 weeks of synbiotic treatment enhanced immune system responses and decreased the occurrence and length of URTIs more efficiently than prebiotics alone. According to Zhang et al. (66), it also improved recovery capacity by lowering HRmax and improving lactate clearance. as combined with oral iron, a synbiotic product dramatically raised blood ferritin levels in female athletes who were iron deficient over the course of 8 weeks as compared to a placebo (63). In addition to beneficial regulation of immuno-neuroendocrine markers (CRH, dopamine, and IL-1β), 1 month of synbiotic intake in competitive athletes resulted in improvements in the quality of sleep, perceived health, and anxiety/stress levels (9). These findings suggest potential advantages of prebiotic and synbiotic interventions although the evidence base is less comprehensive and more diverse than that for probiotics. Direct comparison is limited because the studies assessed several formulations and outcomes, such as gastrointestinal symptoms, immunity, iron status, recuperation, and performance. Furthermore, gains in recuperation or indirect health indicators shouldn't always be taken as proof of improved athletic performance (Table 1).

**Table 1:** Probiotic, prebiotic, synbiotics and post-biotics administration techniques improving muscle performance and gut integrity in athletes.

Intervention (form)	Population (*N*)	Design	Duration	Dosage	Key outcomes	References
Probiotic (*Lactiplantibacillus plantarum* TWK10)	Male and female adults (32)	Double-blind, randomized, controlled trial	28 days	1 × 10^10^ CFU	Improved anaerobic capacity, muscle strength, power, gut flora, and absorption of amino acids	Lee et al. (96)
Probiotic (*Bacillus coagulans*)	Trained males (30)	Crossover, diet-controlled design	7 weeks	1 billion CFU	Decreased muscle injury, enhanced recuperation, and sustained athletic performance after intense resistance training	Jäger et al. (97)
Prebiotic (Inulin–FOS)	Endurance athletes (16)	Randomized controlled trial	8 weeks	16 g/day	Reduced intestinal epithelial damage brought on by exercise without influencing transit or gastrointestinal symptoms	Rauch et al. (62)
Synbiotic	Male university student-athlete (30)	Randomized controlled trial	6 weeks	2 g/d	Improved lactate elimination, decreased frequency and length of URTIs, and no change in VO_2_max	Zhang et al. (66)
Synbiotic supplementation (probiotics + omega-3)	Elite sprint freestyle swimmers (60)	Randomized, double-blind trial	8 weeks	Two capsules per day	Enhanced torque, power, dynamic upper-body strength, and rate of force development	Imanian et al. (65)
Post-biotic (Urolithin A)	Resistance-trained male athletes (20)	Randomized, double-blind, placebo-controlled	8 weeks	1 g/day	Reduced oxidative stress, decreased inflammation, and increased muscular strength and endurance	Zhao et al. (41)
Post-biotic (Urolithin A)	Male soccer players (20)	Pilot randomized controlled trial	6 weeks	1 g/day	Enhanced jump performance, increased aerobic endurance, and maintained antioxidant levels	Monsalve Acevedo et al. (98)

#### 5.2.1 Applicability and gastrointestinal safety in athletes

Evidence from sedentary or healthy persons should be separated from evidence from trained and elite athletes. Intestinal epithelium damage and permeability can be increased by acute, intense exercise, and gut damage may worsen when exercise is done in hot conditions. This establishes a physiologically unique setting in which the effectiveness and gastrointestinal tolerability of therapies targeting the microbiota should be assessed. Consequently, assertions that probiotics, prebiotics, or synbiotics enhance mucosal integrity should be seen as context- and formulation-specific rather than generally relevant to athletes.

Gastrointestinal safety is somewhat reassured by athlete-specific trials, however their effectiveness varies. When compared to a placebo, Bifidobacterium breve Bif195 did not significantly change intestinal permeability, intestinal fatty acid-binding protein, or gastrointestinal symptom scores in well-trained adults; adverse-event numbers were also comparable between groups. However, an exercise challenge increased intestinal permeability by about 100% (67). Few gastrointestinal symptoms were reported in non-elite runners, and there were no differences between the probiotic and placebo groups (68). Galactooligosaccharide treatment decreased the frequency and intensity of gastrointestinal symptoms in elite rugby union players (61). On the other hand, a triple-blind randomized controlled pilot study in recreationally trained endurance athletes found no significant differences between B-GOS supplementation and placebo in terms of exercise-induced gastrointestinal discomfort, anxiety-related measures, wellbeing, stress, recovery, or sleep at the group level, although individual variation was noted (69). Therefore, the available trials did not show a consistent rise in gastrointestinal symptoms with the particular probiotic or prebiotic formulations examined; however, they do not prove that all microbiome-targeted therapies are safe or effective in protecting the barrier under high training loads.

### 5.3 Post-biotics and microbial metabolite supplementation

In microbiome-based nutrition, post-biotics have shown great promise. They constitute bioactive compounds generated during microbial metabolism, such as SCFAs, bacteriocins, exopolysaccharides, cell wall fragments, and other microbial metabolites generated through the fermentation of dietary substrates. When it comes to regulating the gut-muscle axis, post-biotics might offer a reliable substitute for probiotics. Post-biotics have advantages in terms of stability, storage, and safety because they don't require intestinal colonization to perform biological activity like living bacteria do. SCFAs, such as butyrate, propionate, and acetate, are known to play important roles in intestinal metabolism and the regulation of the epithelial barrier. Further experimental research indicates that these metabolites may have an impact on skeletal muscle's inflammatory, metabolic, and mitochondrial processes. However, animal or cell models provide the majority of evidence for direct modulation of NF-κB, HDAC, AMPK, or muscle-repair pathways. The amounts of SCFAs that reach human skeletal muscle following food fermentation are unknown since they undergo significant intestinal use and hepatic metabolism. As a result, it is currently impossible to interpret these pathways as proven ergogenic or anti-catabolic effects in athletes (10, 70, 71).

Apart from SCFAs, urolithin A (UA) has shown promise as a post-biotic for the health of skeletal muscle (104). Four months of standardized UA supplementation increased muscle strength and was linked to clinically significant increases in specific exercise-performance measures in middle-aged people in a randomized, placebo-controlled experiment (72). In a similar vein, Zhao et al. (41) found that resistance-trained male athletes' maximum voluntary isometric contraction and repetitions-to-failure performance were considerably enhanced by UA supplementation (1 g/day for 8 weeks). Despite the fact that these findings indicate the potential functional benefits of UA supplementation, current clinical investigations have mostly assessed physiological and performance outcomes rather than explicitly verifying the molecular pathways suggested in experimental models. Since human trials of urolithin A directly evaluate functional outcomes, they offer a higher degree of evidence than research based only on cellular or animal models. Nevertheless, there are still only a small number of trials that are available, and those that are do include rather particular demographics and supplementation regimens. Their results support potential functional benefits but they do not indicate that the observed improvements were caused by the suggested mitochondrial or microbiome-mediated processes.

### 5.4 Polyphenol–microbiome interactions

**Figure 3:**
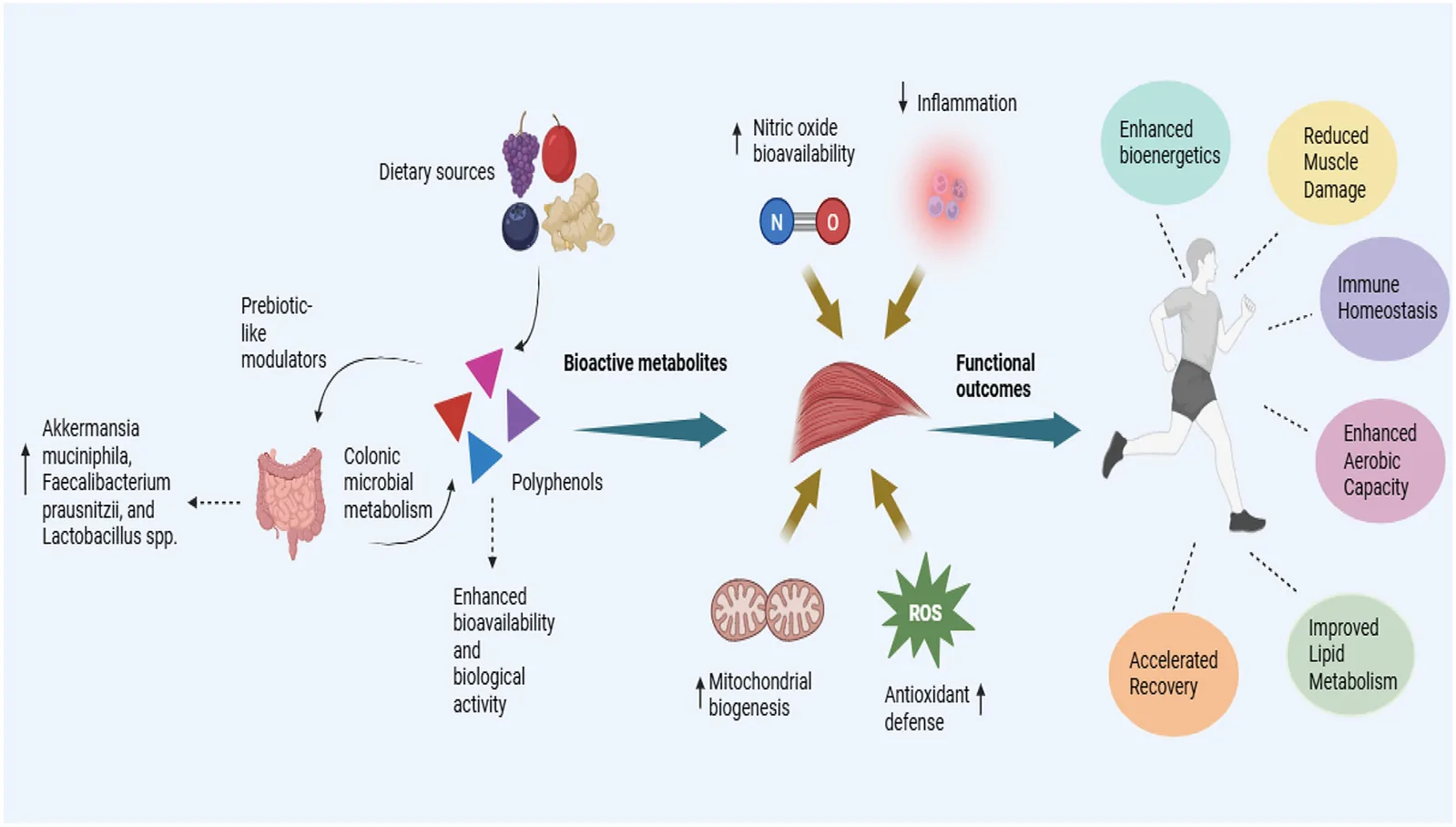
Polyphenol–microbiota relationships and their physiological impacts. *Akkermansia muciniphila, Faecalibacterium prausnitzii*, and Lactobacillus species are among the beneficial taxa that may benefit from dietary polyphenols that undergo intestinal microbial biotransformation into bioactive metabolites. Nitric oxide bioavailability, mitochondrial biogenesis, antioxidant defense, and inflammatory control may all be supported by these alterations, which may have advantages for muscle damage, recovery, aerobic capacity, lipid metabolism, and immunological homeostasis. NO, nitric oxide; ROS, reactive oxygen species.

It has been widely known that polyphenols, a broad class of chemicals originating from plants, have strong anti-inflammatory and antioxidant qualities and can improve sport performance and recuperation. Microbial conversion into bioactive metabolites like phenylpropionic, phenylacetic, and hydroxybenzoic acids is a major factor in their physiological function. These compounds impact skeletal muscle activity and athletic ability through systemic impacts on biosynthesis of mitochondria, antioxidant protection, nitric oxide (NO) accessibility, and inflammatory regulation. Polyphenols work by means of two-way microbiome-host communication. 90–95% of dietary polyphenols are not taken in in the small intestine rather they travel to the colon, where intestinal microbes biotransform them extensively. Complex polyphenolic molecules are broken down into smaller phenolic acids and other byproducts with increased biological value and accessibility by this microbial process (73). Quercetin glycosides, for instance, are converted by intestinal microbes into aglycones and then into phenolic acids like 3,4-dihydroxyphenylacetic acid and 3-hydroxyphenylacetic acid, which have unique anti-inflammatory and metabolic properties (74) (Figure 3).

A significant amount of dietary polyphenols undergo digestive and microbial modification rather than being absorbed in their natural form. Polyphenol structure, dietary matrix, dose, timing, host metabolism, and microbiota composition all have a significant impact on bioavailability. Therefore, it is not possible to presume that quantities utilized in cell experiments will occur in human skeletal muscle upon ingestion of a diet or extract rich in polyphenols. Additionally, polyphenols support beneficial microorganisms like Lactobacillus species, *Faecalibacterium prausnitzii*, and *Akkermansia muciniphila* by acting as prebiotic-like regulators. These microbes and their metabolites may affect AMPK, PGC-1α, oxidative stress, and inflammatory pathways, according to experimental research. However, the majority of human exercise experiments have examined performance, muscular soreness, creatine kinase, or circulating inflammatory markers rather than measuring microbial metabolite exposure and skeletal-muscle pathway activation concurrently. Therefore, the microbiome-mediated activation of AMPK–PGC-1α should be viewed as a suggested mechanism, instead of being a proven explanation for enhanced athletic performance.

A number of polyphenol categories have shown a special significance for sports performance through pathways mediated by the microbiota. Berries and grapes contain resveratrol, a stilbene polyphenol with strong antioxidant and mitochondrial-boosting qualities. In a randomized, double-blind, placebo-controlled study, taking resveratrol daily before plyometric training dramatically decreased muscle soreness and muscle damage indicators (CK, LDH) while improving anaerobic efficiency and muscle strength recovery when compared to a placebo (75). According to Alway et al. (5), resveratrol supplementation in conjunction with training markedly improved muscle endurance, power, mitochondrial number, and fiber hypertrophy.

Berries like sour cherries, blackcurrants, and haskap are rich in anthocyanins, which have been repeatedly proved to improve endurance and exercise recovery. According to a randomized study, consuming tart cherry juice both before and during a strenuous running event sped up the recuperation of muscular function and decreased post-run muscle discomfort as opposed to a placebo (76). In a different study, anthocyanins from New Zealand blackcurrant extract improved time-trial efficiency and fat oxidation during submaximal exertion in trained cyclists, which improved endurance capacity (77). According to Howatson et al. (78), supplementing with Haskap berries also considerably increased endurance running efficiency, extending time to fatigue by 20 s and improving 5 km time-trial performance by about 21 s (~2% improvement) in contrast to a placebo. In trained male and female athletes, anthocyanin intake for 6 weeks significantly raised VO_2_max when compared to a placebo, suggesting improved aerobic capacity (79). Onions and apples contain quercetin, a flavonol that has drawn interest due to its dual activities as an antioxidant and a stimulant of the biosynthesis of mitochondria. In comparison to a placebo, quercetin administration for 7 days improved VO_2_max by 3.9% and extended delay to exhaustion by 13.2% (80). Furthermore, in trained male athletes, quercetin intake, with or without vitamin C, boosted metabolic variables and body composition, such as lean mass, overall energy consumption, and basal metabolic rate. It also demonstrated a pattern toward boosted aerobic capacity (VO_2_max) (81) (Table 2).

**Table 2:** Impact of plant-based polyphenols on muscle damage and performance adaptation.

Intervention (form)	Population (*N*)	Design	Duration	Dosage	Key outcomes	Reference
Resveratrol	Untrained males (36)	Double-blind, placebo-controlled trial	10 days	500–1,000 mg/day	Enhanced anaerobic performance, less pain and damage to the muscles, and quicker recuperation	Huang et al. (75)
Resveratrol	Male participants (8)	Single-blind crossover study	4 days	480 mg per day	Decreased exercise-induced IL-6 response without affecting oxidative damage, fatigue, or endurance performance	Tsao et al. (99)
Blackcurrant extract (anthocyanins)	Female cyclists (16)	Randomized, crossover, double-blind design	7 days	600 mg/day	Enhanced fat oxidation and increased resting plasma NEFA and glycerol concentrations	Strauss et al. (100)
Blackcurrant extract (anthocyanins)	Cyclist (34)	Randomized, double-blind, and placebo-controlled crossover design	One month	900 mg/d	Enhanced cycling time-trial performance, power output, and speed in slower endurance-trained cyclists	Montanari et al. (101)
Haskap berry (anthocyanins)	Male runners (30)	Double-blind, placebo controlled, independent groups design	6 days	6 g/day (~150 mg anthocyanins/ dose)	Enhanced 5 km time-trial performance, time to fatigue, and endurance running performance	Howatson et al. (78)
Quercetin	Male students (60)	Double-blind clinical trial	8 weeks	500 mg/day	Elevated basal metabolic rate, total body water, lean body mass, and total energy expenditure	Askari et al. (81)
Quercetin	Untrained participants (12)	Randomized, double-blind, placebo-controlled, crossover study	7 days	500 mg (Twice daily)	Elevated VO_2_max and ride time to fatigue	Davis et al. (80)
Curcumin	Elite rugby players (10)	Randomized cross-over design	15 days	2 g/day	Decreased the loss of sprint power following exercise and mitigated some aspects of muscle damage	Delecroix et al. (102)
Curcumin	Males (20)	Double-blind crossover	7 days	180 mg/day	Decreased acute inflammation, lessened muscular damage, and accelerated post-exercise recuperation	Tanabe et al. (83)
Green tea extract	Untrained men (20)	Randomized triple blind placebo control study	15 days	500 mg/day	Decreased indicators of muscle injury without impacting oxidative stress or muscular pain.	da Silva et al. (86)
Green tea extract	Male sprinters (16)	Double-blind, randomized, placebo (PL)-controlled crossover study	4 weeks	980 mg/day	Reduced oxidative stress brought on by exercise without enhancing sprint performance or muscle damage	Jówko et al. (84)
Green tea extract	Male students (35)	Randomized, double-blind design	4 weeks	640 mg/day	Improved antioxidant defense and decreased oxidative damage following muscle endurance and strength training	Jówko et al. (103)

The main curcuminoid in turmeric, curcumin, has substantial anti-inflammatory properties, primarily because it is converted to tetrahydrocurcumin by gut microbes. After workout-related muscle damage, curcumin administration substantially decreased felt muscle pain and muscle injury (CK levels) (82). Additionally, the advantageous effects of curcumin administration on exercise recovery were time-dependent. Before-exercise consumption decreased acute inflammatory reactions, whereas after-exercise consumption decreased muscle damage and discomfort, accelerating functional recovery (83). Interactions between gut microorganisms and catechins, especially epigallocatechin gallate (EGCG), result in the production of metabolites with increased antioxidant properties. According to some research, multi-week green tea extract suppressed CK following sprint protocols and decreased oxidative stress indicators in sprinters and other trained individuals. These findings may help high-intensity athletes recuperate (84). Another study found that supplementing trained male athletes with green tea extract for 15 days preserved neuromuscular performance throughout successive exercise sessions while reducing oxidative stress and impairment of muscles (85). According to a similar research, green tea extract's catechins improved post-exercise muscle recuperation by reducing exercise-associated muscle injury (86). Yet, given that certain green tea preparations contain caffeine, which may independently improve alertness, substrate utilization, and exercise performance, these outcomes should be interpreted cautiously (87, 88). Therefore, in order to differentiate between catechin-mediated antioxidant effects and caffeine-driven ergogenic responses, new research should explicitly state caffeine concentration and, whenever feasible, employ decaffeinated or caffeine-matched control extracts. The clinical findings generally indicate that various polyphenol-rich therapies may improve specific measures of exercise recovery or performance although the data is not consistent across polyphenol classes or outcomes. It is challenging to generalize the claimed effects of resveratrol, anthocyanins, quercetin, curcumin, and green tea extracts because these substances have been studied in various populations and exercise models. Furthermore, the majority of studies evaluated oxidative responses, performance, soreness, or muscle-damage markers without explicitly proving that modifications in the gut microbiota or microbial metabolites mediated these advantages. As of right now, there is insufficient data to determine what athletes should eat, how much, or when in order to consistently increase their performance. The regimens outlined in this analysis are not evidence-based recommendations, but rather protocols assessed in separate studies. Direct comparisons between intervention courses are impossible due to variations in the athlete demographic, training status, study length, dietary background, and outcome selection. Replicated, athlete-specific dose-response and timing studies for each intervention independently are therefore necessary for practical recommendations.

## 6 Future directions

Future research on the microbiome-based nutrition and gut-muscle axis should focus on quantifiable, athlete-relevant biomarkers rather than broad taxonomic characterization. Integrated panels combining metagenomics, metabolomics, lipidomics, inflammatory markers, gut-barrier markers, and muscle-performance outcomes should be prioritized. Microbial functions, circulating metabolites, and sport-specific physiology may better account for inter-individual variations in endurance, strength, recovery, and fatigue, according to recent human investigations in athletes (89). The creation of biomarker profiles that can determine when the gut ecology favors adaptation or when it indicates excessive training stress is a crucial avenue.

The significant interindividual variance among athletes presents another difficulty in putting microbiome-based nutrition into practice because a universal microbiome-targeted intervention is unlikely to be equally successful in all individuals. Sport discipline, training exposure, food consumption, and other athlete-specific factors all influence the microbiome profiles of athletes (90, 91). A multi-cohort study of athletes participating in wrestling, rowing, and aerobics showed that athletes had gut microbiota specific to their sport and found microbial subgroups linked to anaerobic performance, inflammation, and diet (21).

An athlete's intestinal ecology may also be impacted by their training load. In highly skilled rowers, gut microbiome traits, short-chain fatty acid concentrations, stool frequency, and diet quality varied between periods of high and low training load, but intake of carbohydrates, fat, protein, and fiber stayed constant (109). Collectively, these results suggest that microbiome data should not be evaluated in isolation, but rather in the context of the athlete's sport, nutrition, and training. Therefore, sport discipline, training volume and intensity, habitual food intake, gastrointestinal symptoms, baseline microbiome characteristics, and the nutritional intervention's composition should all be consistently documented in future research. It is important to evaluate individual reactions in addition to group-level findings. Probiotic evidence for athletes is still inconsistent. Potential increases in inflammatory biomarkers and sports performance were found in a systematic evaluation of nine randomized clinical trials; however, the majority of the included studies were deemed to have a high risk of bias, which limited the findings' repeatability (110). Similar findings were found in an exploratory randomized, double-blind, placebo-controlled trial involving healthy male athletes who were eating a high-protein diet; however, *post hoc* analyses indicated that probiotic responsiveness may be influenced by baseline microbiome composition. These results are still hypothesis-generating and need to be confirmed in sufficiently powered trials with predetermined objectives before being converted into customized nutritional approaches (111).

Another significant barrier to microbiome research is methodological variation. Microbiome profiles can be influenced by variations in study design, participant selection, sample collection and processing, sequencing techniques, and bioinformatic pipelines, all of which can lead to conflicting results between studies. Increased methodological uniformity and open reporting will boost repeatability and fortify the interpretation of results pertaining to the microbiome (92, 93).

Adequately powered, double-blind, randomized, placebo-controlled trials should be carried out specifically in trained and elite athletes. Instead of being considered as a unified microbiome-based intervention, probiotics, prebiotics, synbiotics, post-biotics, and polyphenols should be assessed as distinct intervention classes utilizing precisely defined formulations, dosages, durations, and timing schedules. To increase comparability between studies, it is also necessary to standardize or closely evaluate gastrointestinal tolerance, training load, background supplements, adherence, and habitual diet. It is now impossible to identify which intervention, dose, duration, or timing strategy consistently enhances athletic performance because the evidence is still inconsistent among intervention classes, formulations, and athlete populations.

Fecal short-chain fatty acids, lactate-using taxa, bile-acid profiles, microbial lipid mediators, plasma amino acid metabolites, zonulin, I-FABP, LBP, cytokines, creatine kinase, and recovery-related metabolomic patterns are examples of potential markers (34). Instead of just using single time-point comparisons, these biomarkers should be evaluated longitudinally throughout training stages, competition, tapering, injury healing, and dietary treatments. New technologies will play a key role in this advancement. Real-time connections between nutrition, microbiota, training load, sleep, gastrointestinal symptoms, and muscle function can be made using shotgun metagenomics, strain-level sequencing, metabolomics, single-cell immunological profiling, wearable sensors, digital dietary tracking, and machine learning models.

Instead of depending only on microbiome composition and performance outcomes, future randomized human studies should quantify every step of the suggested gut–muscle relationship to enhance causal inference. Assessment of strain-specific persistence, validated metabolomic or stable isotope-tracing methods to differentiate intervention-derived metabolites from background microbial metabolism, concurrent assessment of intestinal and circulating metabolite exposure, skeletal-muscle molecular endpoints (such as PGC-1α expression, mitochondrial function, and AMPK phosphorylation), and mediation analyses connecting these biological measurements with predetermined exercise outcomes should all be included in study designs. It would be clear from such integrated designs whether metabolites derived from probiotics directly mediate physiological changes or merely accompany them.

Additionally, complementary mechanistic models will be needed. It may be possible to ascertain whether microbial metabolites directly affect glucose uptake, mitochondrial function, neuromuscular signaling, or post-exercise repair using germ-free mice, fecal microbiota transplantation, muscle-cell systems, gut organoids, and gut–muscle co-culture platforms (94, 95). Practically speaking, microbiome-targeted therapies should currently be implemented individually rather than as general ergogenic methods. Prior to implementation, sports nutritionists should choose interventions based on the particular strain or formulation assessed in athlete studies and take into account the athlete's training load, regular diet, gastrointestinal complaints, and performance objectives. To assess individual responsiveness during supplementation, gastrointestinal tolerance, adherence, training interruption due to illness, recuperation, and sport-specific performance outcomes should be tracked. Before being included in competitive nutrition programs, novel microbiome-targeted supplements should be assessed during regular training because responses vary among athletes and intervention types (61, 67, 68).

## 7 Conclusion

A biologically feasible paradigm that connects microbial activity with muscle metabolism, recuperation, and exercise adaption is the gut–muscle axis. Experimental models show that the delivery of microbial metabolites or modification of the gut microbiota can affect muscle-related outcomes under controlled circumstances. Human research, on the other hand, mostly document correlations or functional enhancements after microbiome-targeted therapies without determining the entire molecular process. Seldom have probiotic durability, strain-specific metabolite synthesis, systemic metabolite exposure, and downstream skeletal-muscle signaling all been assessed at the same time. Therefore, microbiome-based nutritional strategies should be considered as promising methods, however human confirmation of the postulated molecular processes linking microbiome manipulation to improved athletic performance is still pending.
